# Plumbagin: A Promising In Vivo Antiparasitic Candidate against *Schistosoma mansoni* and In Silico Pharmacokinetic Properties (ADMET)

**DOI:** 10.3390/biomedicines11092340

**Published:** 2023-08-22

**Authors:** Lucas M. N. Silva, Wilza W. M. França, Victor H. B. Santos, Renan A. F. Souza, Adriana M. Silva, Emily G. M. Diniz, Thierry W. A. Aguiar, João V. R. Rocha, Mary A. A. Souza, Wheverton R. C. Nascimento, Reginaldo G. Lima Neto, Iranildo J. Cruz Filho, Eulália C. P. A. Ximenes, Hallysson D. A. Araújo, André L. Aires, Mônica C. P. A. Albuquerque

**Affiliations:** 1 Programa de Pós-Graduação em Ciências Farmacêuticas, Departamento de Ciências Farmacêuticas, Universidade Federal de Pernambuco, Recife 50740-520, PE, Brazil; lucas.matheusnascimento@ufpe.br (L.M.N.S.); victorhsantos7@gmail.com (V.H.B.S.); renan.fernandes@ufpe.br (R.A.F.S.); eulaliaximenes@yahoo.com.br (E.C.P.A.X.); monica.aalbuquerque@ufpe.br (M.C.P.A.A.); 2 Instituto Keizo Asami, Universidade Federal de Pernambuco, Recife 50740-465, PE, Brazil; wilza.franca@ufpe.br (W.W.M.F.); adriana.msilva8@ufpe.br (A.M.S.); emilygabriele1999@gmail.com (E.G.M.D.); thierry.wesley@ufpe.br (T.W.A.A.); jvictoritinto@gmail.com (J.V.R.R.); wheverton.nascimento@ufpe.br (W.R.C.N.); hallysson.douglas@ufpe.br (H.D.A.A.); 3 Programa de Pós-Graduação em Medicina Tropical, Departamento de Medicina Tropical Universidade Federal de Pernambuco, Recife 50670-420, PE, Brazil; reginaldo.limant@ufpe.br; 4 Departamento de Bioquímica, Universidade Federal de Pernambuco, Recife 50670-420, PE, Brazil; 5 Programa de Pós-Graduação em Morfotecnologia, Departamento de Histologia e Embriologia, Universidade Federal de Pernambuco, Recife 50670-420, PE, Brazil; profmaryaranda@gmail.com (M.A.A.S.); iranildo.cruz@ufpe.br (I.J.C.F.); 6 Centro de Ciências Médicas—Área Acadêmica de Medicina Tropical, Universidade Federal de Pernambuco, Recife 50670-901, PE, Brazil; 7 Departamento de Antibióticos, Universidade Federal de Pernambuco, Recife 50670-901, PE, Brazil

**Keywords:** naphthoquinone, plumbagin, schistosomicidal, in vivo assay *Schistosoma mansoni*, histopathology

## Abstract

Schistosomiasis, a potentially fatal chronic disease whose etiological agents are blood trematode worms of the genus *Schistosoma* spp., is one of the most prevalent and debilitating neglected diseases. The treatment of schistosomiasis depends exclusively on praziquantel (PZQ), a drug that has been used since the 1970s and that already has reports of reduced therapeutic efficacy, related with the development of *Schistosoma*-resistant or -tolerant strains. Therefore, the search for new therapeutic alternatives is an urgent need. Plumbagin (PLUM), a naphthoquinone isolated from the roots of plants of the genus *Plumbago*, has aroused interest in research due to its antiparasitic properties against protozoa and helminths. Here, we evaluated the in vivo schistosomicidal potential of PLUM against *Schistosoma mansoni* and the in silico pharmacokinetic parameters. ADMET parameters and oral bioavailability were evaluated using the PkCSM and SwissADME platforms, respectively. The study was carried out with five groups of infected mice and divided as follows: an untreated control group, a control group treated with PZQ, and three groups treated orally with 8, 16, or 32 mg/kg of PLUM. After treatment, the Kato–Katz technique was performed to evaluate a quantity of eggs in the feces (EPG). The animals were euthanized for worm recovery, intestine samples were collected to evaluate the oviposition pattern, the load of eggs was determined on the hepatic and intestinal tissues and for the histopathological and histomorphometric evaluation of tissue and hepatic granulomas. PLUM reduced EPG by 65.27, 70.52, and 82.49%, reduced the total worm load by 46.7, 55.25, and 72.4%, and the female worm load by 44.01, 52.76, and 71.16%, for doses of 8, 16, and 32 mg/kg, respectively. PLUM also significantly reduced the number of immature eggs and increased the number of dead eggs in the oogram. A reduction of 36.11, 46.46, and 64.14% in eggs in the hepatic tissue, and 57.22, 65.18, and 80.5% in the intestinal tissue were also observed at doses of 8, 16, and 32 mg/kg, respectively. At all doses, PLUM demonstrated an effect on the histopathological and histomorphometric parameters of the hepatic granuloma, with a reduction of 41.11, 48.47, and 70.55% in the numerical density of the granulomas and 49.56, 57.63, and 71.21% in the volume, respectively. PLUM presented itself as a promising in vivo antiparasitic candidate against *S. mansoni*, acting not only on parasitological parameters but also on hepatic granuloma. Furthermore, in silico, PLUM showed good predictive pharmacokinetic profiles by ADMET.

## 1. Introduction

In 2021, about 251.4 million people were diagnosed with schistosomiasis, a chronic infectious, parasitic disease, and the world’s second most important parasitic disease in terms of socioeconomic and public health impacts [1]. Schistosomiasis is prevalent in 78 countries and territories in tropical and subtropical regions, where approximately 800 million people live at constant risk of infection [1,2]. The etiological agents are blood helminths of the genus *Schistosoma* spp. [3], with greater epidemiological importance for *Schistosoma mansoni* due to its high prevalence, being the only species found in the Americas, including Brazil [4]. Populations with low political and economic visibility are the most affected and constantly exposed to infection and reinfections, since they inhabit areas with scarce or lacking basic sanitation, health care, diagnosis, early treatment, and health education actions, all essential conditions to interrupt the transmission cycle [5,6,7,8,9,10]. 

Schistosomiasis mansoni is a potentially fatal infection, responsible for about 12,000 deaths annually. However, these figures are likely underestimated and need to be reassessed [1]. The morbidity of the infection is a consequence of the granulomatous inflammation triggered by the reaction to the antigens secreted by the eggs retained in the intestinal and, especially, in hepatic tissues. In the course of the granulomatous inflammatory process may be observed the occurrence of portal hypertension, hepatosplenomegaly, ascites, fibrosis, esophageal varices, and hematemesis [11,12,13]. 

**Figure 1:**
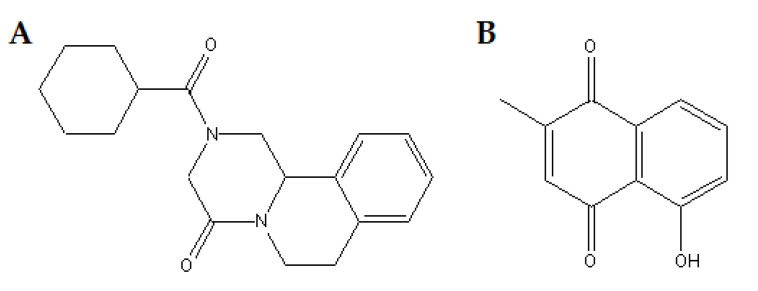
Chemical structure of (**A**), praziquantel (PZQ) and (**B**), plumbagin (PLUM).

There is still no safe vaccine, so the current and only strategy to reduce the prevalence and incidence of schistosomiasis depends solely on praziquantel (PZQ) (Figure 1A), a drug available since the 1970s [14,15]. Nowadays, PQZ is available in its racemic form, mixtures of two enantiomers of a chiral molecule. The (R)-PZQ conformation is what has anthelmintic activity, whereas (S)-PZQ is inert, responsible for the bitter taste, which causes rejection and difficulty in treatment, especially in children [14]. Despite the high rate of parasitological cure only against adult worms of all species of *Schistosoma* spp., few side effects, and low cost, PZQ has no prophylactic action, does not prevent reinfection and cannot reverse the histopathological damage that causes irreversible sequelae (mainly in the liver) [16,17]. In addition, there is reported resistance both in the field and experimentally induced on *Schistosoma* spp. strains, especially in endemic areas where mass administration is recurrent, a scenario that signals a future collapse in the treatment and control of schistosomiasis [18,19]. 

The lack of therapeutic alternatives for schistosomiasis is worrying, since it is classified as a neglected tropical disease by the pharmaceutical industry and development agencies and continues to expand [20,21]. Thus, scientific and medical communities, in line with international policies, encourage the search for new schistosomal drug candidates [22,23]. The investigation of plant extracts and secondary metabolites as promising antischistosomal compounds is an important strategy for the development of drug candidates. Some of these compounds present known biological, pharmacological, and biosafety properties, and therefore, may be repositioned as new biological and therapeutic targets, in order to generate cost- and time-savings [24,25,26,27,28]. 

Quinones are divided into three groups based on their chemical structures: anthraquinones, benzoquinones, and naphthoquinones, which have a naphthalene ring [29,30]. Naphthoquinones are known as a wide and varied family of metabolites produced by several species of plants, fungi, bacteria, arthropods, and animals [31,32]. These are known to present biological properties such as antibacterial [33,34], anticancer [35], antifungal [34], anti-inflammatory [36], and cardioprotective, hepatoprotective, and neuroprotective [37]. In addition, these compounds have potential in controlling pests and vectors [38,39], as a larvicide, and a mosquitocide [40]. Naphthoquinones have been highlighted as a promising antiparasitic agent against *Leishmania* spp. [41,42,43], *Trypanosoma cruzi* [41], *Plasmodium* spp., [44], *Toxoplasma gondii* [45], *Toxocara canis* [46,47], *Hymenolepes nana* [48], and *S. mansoni* [49,50]. 

Among naphthoquinones, plumbagin (PLUM) (5-hydroxy-2-methyl-1,4-naphthoquinone) is a polycyclic aromatic hydrocarbon (Figure 1B) that can be naturally isolated from various parts of plants of the families Plumbaginaceae, Ebenaceae, and Droseraceae, and has a broad spectrum of biological and pharmacological properties including activation of apoptosis, antioxidant, anti-inflammatory [51] anticancer [52], antibacterial [53], and antifungal [29,54]. Regarding the antiparasitic potential, extracts obtained from *Plumbago auriculata*, *Plumbago zeylanica* L., and *Plumbago indica* L., all rich in plumbagin, demonstrated action against *Trypanosoma cruzi*, *Plasmodium falciparum*, and *Carmyerius spatiosus*, respectively [55,56,57]. Furthermore, isolated plumbagin was able to induce programmed cell death (PCD) *Leishmania* promastigotes through elevation of reactive oxygen species (ROS), disruption of intracellular calcium homeostasis, and mitochondrial depolarization and by acting as an inhibitor of trypanothione redutase [42,58]. PLUM was also able to reduce the parasitemia of *Plasmodium* spp. [59] and *T. cruzi* [42,60]; on *Fasciola gigantica* [61] and *Paramphistomum* [62] it caused damage to the integument and death. Furthermore, PLUM also damaged the integument and caused muscle contraction, mitochondrial damage, protein disorganization and lipid peroxidation due to increased ROS and H_2_O_2_ against *S. mansoni* in vitro and in vivo [63,64,65].

Thus, given the antiparasitic effect of PLUM, the present study aimed to explore its action when administered orally against adult worms of *S. mansoni*, through the evaluation of the parasite load, the load of eggs in the feces, and the hepatic and intestinal tissues, oviposition pattern, and the histopathological and histomorphometric evaluation of tissue and hepatic granulomas.

## 2. Materials and Methods

### 2.1. Drugs

Praziquantel (2-(cyclohexyl carbonyl)-1,2,3,6,7-11b-hexahydro-4H-pyrazine [2,1-a]isoquinolin-4-one (C_19_H_24_N_2_O_2_, molecular weight 312.41, purity ≥ 98%)) (https://www.sigmaaldrich.com/BR/pt/substance/praziquantel3124155268741) (accessed on 8 May 2022) and plumbagin (5-hydroxy-2-methyl-1,4-naphthoquinone (C_11_H_8_O_3_, molecular weight 188.18)) from *Plumbago indica* (https://www.sigmaaldrich.com/EN/en/product/sigma/p7262) (accessed on 8 May 2022) and all analytical and culture grade reagents were obtained from Sigma Chemical Co., St. Louis, MO, USA.

### 2.2. Ethical and Animal Considerations

All experimental protocols were approved by the Animal Experimentation Ethics Committee of the Biosciences Center of the Federal University of Pernambuco (CEEA/UFPE) (nº 23076.011182/2018-01). Fifty female *Swiss Webster* mice (35 days old, ~30 ± 2 g) were supplied and maintained in the vivarium of the Institute Keizo Asami UFPE (iLIKA-UFPE) according to standardized rearing conditions (23 ± 2 °C, 40–50% humidity, and 12 h light/dark photoperiod with water and food, Labina^®^, ad libitum). The strains of *Biomphalaria glabrata* and *S. mansoni* (BH strain; Belo Horizonte—MG, Brazil) are maintained for successive generations in the mollusk at the Department of Parasitology of the Academic Area of Tropical Medicine at UFPE. The snails are kept in plastic tanks (50 × 23 × 17 cm) with filtered and dechlorinated water (20 L), changed weekly, fed daily with fresh lettuce leaves (*Lactuca sativa* L.), at a temperature between 25 ± 2 °C and with a 12 h light/dark cycle.

#### 2.2.1. Infection of *Biomphalaria glabrata* with *Schistosoma mansoni* Miracidia and Cercarial Shedding

**Figure 2:**
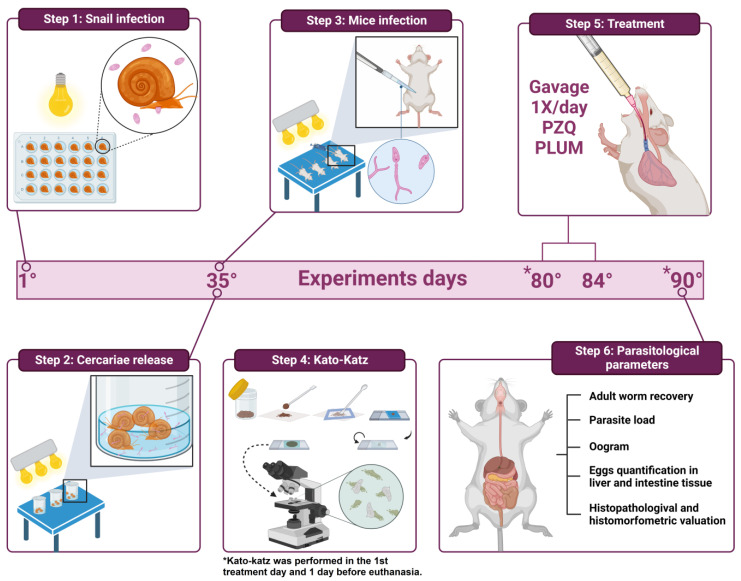
Design of the experiment. Step 1—Experimental infection of *B. glabrata* snails with *S. mansoni* miracidia (BH strain). Step 2—Obtaining *S. mansoni* cercariae. Step 3—Percutaneous infection in mice with cercarial suspension. Step 4—Kato–Katz method for counting eggs eliminated in feces performed on the 45th day after infection, before starting the therapeutic intervention, and on the 55th day after infection, before euthanasia of the experimental groups. Step 5—Therapeutic intervention with PZQ or PLUM during five consecutive days (45th to 55th day after infection) orally by gavage. Step 6—Evaluation of the therapeutic intervention through the parasitological parameters of recovery and quantification of the parasitic load, oogram, quantification of the load of eggs deposited in the hepatic and intestinal tissues, and histopathological and histomorphometric evaluation of hepatic schistosomal granulomas.

*S. mansoni* eggs were obtained from the feces of experimentally infected mice. The fecal material was collected and processed according to the method of Araújo et al. [66]. After successive washes, the sediment was distributed in Petri dishes and exposed to artificial light (60 W, Lightex, model A5570) for the hatching of the miracidia. With the aid of a stereoscopic microscope, all snails were individually infected with five miracidia in 24-well culture plates (TPP-Techno Plastic Products, Trasadingen, Switzerland) and exposed to artificial light for 4 h (Figure 2, step 1). After this period, the snails were transferred to plastic tanks in an environment protected from light. After 35 days of infection, snails (n = 80) were exposed for 2 h to artificial light (60 W) to release cercariae (Figure 2, step 2).

#### 2.2.2. Infection of Mice and Experimental Groups

All mice (n = 50) were anesthetized with intramuscular administration of ketamine-xylazine (100–200 mg/kg + 5–16 mg/kg) and percutaneously infected (Figure 2, step 3) [67] with a cercarial suspension (n = 50 cercariae). Subsequently, the mice were randomly distributed into five experimental groups (G1–G5) with 10 animals each, as described below.

- G1 = animals that received only sterile saline solution;
- G2 = animals treated with PZQ;
- G3, G4, and G5 = animals treated with PLUM.

Treatment at the curative dose with PZQ (G2) was 50 mg/kg/day [50]. Groups G3, G4, and G5 were treated with PLUM at doses of 8, 16, or 32 mg/kg/day, respectively. The dose of PLUM was established based on studies of its cytochrome P450 modulating activity, acute and subacute toxicity, and pharmacokinetic studies [68].

Therapeutic intervention with PZQ or PLUM was performed for five consecutive days starting on the 45th day after infection. PZQ was resuspended in 2% Cremophor and solubilized in sterile saline, while PLUM was previously diluted in 0.5% dimethylsulfoxide (DMSO) in sterile saline and both administered orally by gavage (Figure 2, step 5) to a final volume of 300 µL. Mice free of therapeutic intervention were kept in the same conditions of creation and manipulation, receiving saline solution (300 µL) from the 45th to the 49th day of infection. Before performing the treatment, all groups fasted for 30 min. All groups were euthanized by cervical dislocation on the 55th day of infection.

### 2.3. Parasitological Parameters (Figure 2 Step 6)

#### 2.3.1. Eggs per Gram of Feces

About the parasitological parameters (Figure 2, step 6), to confirm the homogeneity of the infection, the count of eggs eliminated in the feces was carried out on the 45th day after the infection, before starting the therapeutic intervention. On the 55th day after infection, a new count of eggs eliminated in the feces was performed to assess the impact of the treatment. Briefly, individual fecal samples were collected from all mice and processed according to the Kato–Katz method (Figure 2, step 4) [69]. The analysis was performed in duplicate and the results are expressed as the average number of eggs/gram of feces.

#### 2.3.2. Worm Recovery

Worms were recovered from the hepatic portal system and mesenteric vessels by perfusion with sterile saline (0.9% *w*/*v* NaCl) as described by Smithers and Terry [70]. The worms were quantified and classified into couples and males. The percentage reduction in the number of worms after treatment was calculated according to the equation: % reduction = C − V/C × 100, where C = the mean number of worms recovered from infected, untreated animals and V = the mean number of worms recovered from treated animals [71].

#### 2.3.3. Oogram Pattern

Fragments of the middle portion of the small intestine (~3 cm in length) were removed and used to evaluate the maturation development of *S. mansoni* eggs according to Pellegrino et al. [72]. Briefly, intestinal tissue samples were cut lengthwise, washed in saline, gently dried on filter paper, and then compressed between slides and cover slip to obtain the thin preparation. Then, the fragments were analyzed under an optical microscope (10×) for classification and percentage counting of the stages of *S. mansoni* eggs: immature (eggs in 1st, 2nd, 3rd, and 4th stages), mature or dead.

#### 2.3.4. Quantification of Eggs in the Liver and Intestinal Tissue

Liver and intestinal tissue were processed separately by the potassium hydroxide digestion technique (5% KOH), as described by Cheever et al. [73]. The results were expressed as an average number of eggs per gram of tissue.

#### 2.3.5. Histomorphological and Histomorphometric Evaluation of Hepatic Granulomas 

Liver tissue samples were fixed in buffered formalin (10%) and processed for paraffin embedding to obtain histological sections (5 µm), which were stained with hematoxylin and eosin and used for histopathological evaluation using an optical microscope Labomed Lx400 microscope (Labomed Inc. Los Angeles, CA, USA). 

For the evaluation of the numerical density of granulomas, 20 random fields per histological sample of each animal were used for the average count of granulomas, according to Formula (1). Each field measured 12,234 μm^2^ [74].

Considering that schistosomal granuloma is a slightly spherical three-dimensional inflammatory condition, the volume of the granuloma was calculated as follows: twenty granulomas containing a single central egg were randomly used to determine the average diameter, using Formula (2). The radii of the granulomas as well as their diameters were measured and used to calculate the volume according to Equation (3) [74]:

(1) $$\;Average\;number\;of\;granulomas=\frac{\sum_{}^{}granulomas}{number\;of\;fields\;analyzed}$$

(2) $$\;Average\;diameter\;of\;granuloma=\frac{\Sigma \;two\;perpendicular\;diameters\;transmiracidial}{2}$$

(3) $$\;Granuloma\;volume=\frac{4\pi r³}{3}$$

Images were obtained using an optical microscope Labomed Lx400 microscope (Labomed Inc., Los Angeles, CA, USA) connected to a digital camera (Nikon^®^) and a computer system (Motic Images Plus 2.0 MLTM). All analyses were performed by two different pathologists.

### 2.4. In Silico Evaluation of Pharmacokinetic Parameters: Absorption, Distribution, Metabolism, Excretion, Toxicity (ADMET) and Prediction of Oral Bioavailability

The prediction of the in silico parameters of absorption, distribution, metabolism, excretion, toxicity (ADMET), and bioavailability was performed according to the methodology proposed by Pires et al. [75] and Diana et al. [76]. Initially, the SMILES codes (Simplified Molecular Input Line Entry Specification) and the bioavailability profile (Lipinski and Veber rules) were obtained for PLUM and PZQ through the SwissADME platform (http://www.swissadme.ch/index.php) (accessed on 8 May 2022) (the codes were obtained by designing the structures). Then, the SMILES codes were inserted into the pkCSM platform (http://biosig.unimelb.edu.au/pkcsm) (accessed on 8 May 2022) to perform the prediction of the ADMET parameters. The platforms used are free.

### 2.5. Statistical Analyzes

GraphPad Prism 5.0 (GraphPad Software, San Diego, CA, USA) was used for statistical analysis. Results are expressed as mean ± standard deviation (SD). Analysis of variance (ANOVA) was used to compare different experimental groups. When ANOVA revealed a significant difference, the Bonferroni post hoc test was used to identify the difference between groups. Differences were considered significant when *p* < 0.05.

## 3. Results and Discussion

### 3.1. Effect of PLUM on Schistosoma mansoni

**Figure 3:**
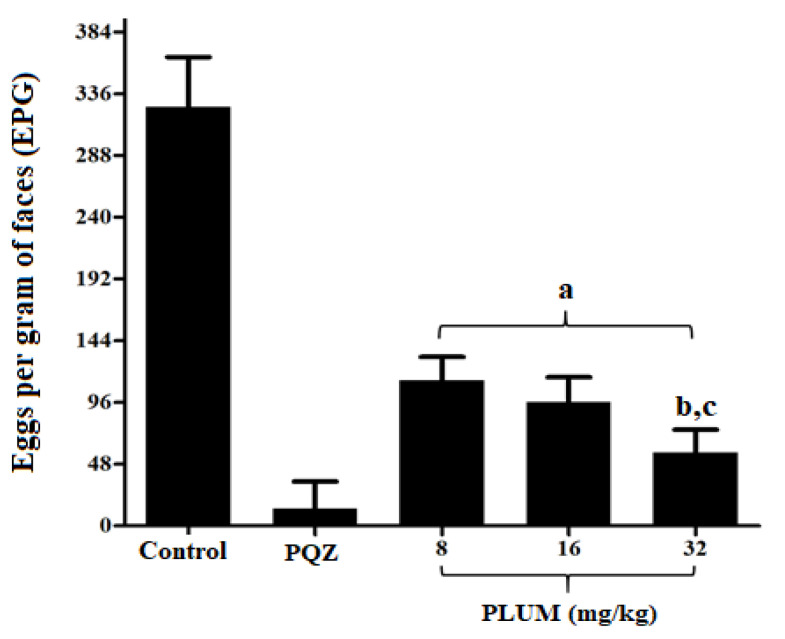
Effects of plumbagin (PLUM) and praziquantel (PZQ) on the number of eggs per gram of feces of mice infected with *S. mansoni*. ^a^ *p* < 0.001 compared to control and PZQ groups; ^b^ *p* < 0.001 compared to 8 mg/kg group; and ^c^ *p* < 0.01 compared to 16 mg/kg group.

On the 45th day after infection, the egg load estimated by the Kato–Katz method showed no significant difference (*p* < 0.5) between all experimental groups, ranging from 285.3 ± 47.16 to 289.7 ± 53.81 eggs per gram of feces (EPG), ensuring uniformity in the infection before starting therapeutic intervention with PLUM or PZQ. The Kato–Katz technique is the gold standard and therefore recommended by the World Health Organization for the diagnosis and determination of the intensity of infection of geohelminths and intestinal *Schistosoma* spp. species, since it does not require extensive infrastructure and is easy to perform, providing a standardized reading in eggs estimated per gram of feces and is low cost, proving to be as sensitive as other techniques [69,77,78]. In this context, the technique is widely used in epidemiological surveys and the monitoring of infections, in addition to being recommended for the evaluation of post-treatment with new schistosomicidal drugs in research [77,79]. To evaluate the effect of PLUM on the number of EPG after the therapeutic intervention, a new egg count was performed after 55 days of infection, before euthanasia. According to Figure 3, it is possible to observe that treatment with PLUM significantly reduces (*p* < 0.001) the number of eggs eliminated in the feces by 65.27, 70.52, and 82.49% for doses of 8, 16, and 32 mg/kg, respectively, when compared to the control group. The 32 mg/kg dose was more effective than the 8 (*p* < 0.001) and 16 mg/kg (*p* < 0.01) doses, the latter two with no statistical difference between them. Our results are promising as candidates for schistosomicidal drugs that lead to the reduction in the release of eggs in feces; consequently, contributing to the restricting environmental contamination and interruption of the *S. mansoni* biological cycle.

In the treatment with PZQ, the EPG reduction was 95.9% when compared to the control group, in line with the results of Cruz et al. [80]. The reduction achieved in both treatments, PLUM or PZQ, can be attributed to the schistosomicidal action on whole worms and/or female worms. Here, treatment with PLUM at doses of 8, 16, and 32 mg/kg reduced (*p* < 0.001) the total worm burden by 46.7, 55.25, and 72.4%, and the female worm burden by 44.01, 52.76, and 71.16%, respectively. As shown in Table 1, the dose of 32 mg/kg showed a significant difference with the doses of 8 and 16 mg/kg for the burden of total worms and female worms. In this context, El-Beshbishia et al. [81], demonstrated that artemisinin associated with naphthoquinone phosphate (CO-ArNp) reduced the total worm load by 74.71% at a dose of 500 mg/kg. A fifteen times smaller reduction was also observed with PLUM treatment at 32 mg/kg. In order to explore naphthoquinones against *S. mansoni*, Aires et al. [50] reports that β-lapachone at a dose of 50 mg/kg reduced the total and female worm burden by 41%. Additionally, Kapadia et al. [82] demonstrated that menadione (vitamin K3) at doses of 40 or 400 mg/kg reduced the total worm burden by 48.57 and 61.90%, with no difference between doses. In this sense, when considering treatment with naphthoquinones, the dose selection should take into account the significant biological effect and toxicological tolerance [83]. Unexpectedly, the effect of PLUM on infected mice with the *S. mansoni* Egyptian strain (John Bruce Egyptian strain) was recently explored by Bakery et al. [65]. In this study, the mice were treated with intraperitoneal injections at a total dose of 20 mg/kg body weight divided equally into four injections, two injections/week for consecutive weeks, the treatment started after 42 days of infection. For this experimental design, the reduction was 60.95% for total worms and 59.88% for female worm load. Our study seeks to contribute to the schistosomicidal potential of PLUM against *S. mansoni*, strain BH (Belo Horizonte—Minas Gerais, Brazil) and using different doses administered orally daily, since it is known that different strains of *S. mansoni*, administration routes, and drug dosages may result in different responses [81,82,84].

The 50 mg/kg dose of PZQ reduced the total and female worm burden by 97%. Despite being the only drug used since the 1970s, the exact mechanism of action of PZQ is still unknown. However, it is known that in the *Schistosoma* spp. species, PZQ unbalances the homeostasis of the *β* subunit of the Ca^2+^ channels in adult worms, inducing rapid and permanent spasmodic muscle contraction and paralysis, followed by integumentary changes and the subsequent death of the worm [85]. Regarding the mechanism of action of PLUM on *S. mansoni*, its effect is attributed to the generation of reactive oxygen species (ROS) via NAD(P)H: quinone oxidoreductase 1 and the increase in H_2_O_2_, interfering in enzymes and proteins of the *S. mansoni*, reducing the glutathione reserve by the oxidation of GSH to GSSG, and resulting in lipid peroxidation and protein disorganization, thus affecting the worm’s ability to protect itself from free radicals, resulting in its death [63,65,86,87]. Furthermore, PLUM has an inhibitory activity on thioredoxin glutathione reductase (TGR), an enzyme that contains selenium and is essential for the survival of *S. mansoni* in the definitive host, being a promising target for drugs, since it is specific to the parasite and absent in its host [65,88,89].

The oogram is considered an important parameter in searching for new schistosomicides [90,91,92]. Corroborating this idea, Aires et al. [50] and El-Beshbishia et al. [81], who have been exploring the naphthoquinones CO-ArNp and *β*-lapachone, respectively, showed that PLUM had no significative effect on the oviposition pattern, considering that all instars evolutionary patterns of *S. mansoni* eggs were visualized. However, PLUM modified the oviposition pattern, since intestinal tissue samples showed a significant reduction in immature eggs and an increase in dead eggs when compared to the control group (*p* < 0.001) (Table 1).

The hypothesis for the reduction in immature eggs can be, in part, attributed to the effect of PLUM on reducing the fecundity of couples, male or female residuals, while the increase in dead eggs can be due to its ovicidal effect, similar to the effect of PZQ [93,94]. Part of the eggs that are not eliminated with the feces are embolized and deposited in the tissues, especially the liver and intestines. The egg in the tissue induces a granulomatous inflammatory response, the main cause of morbidity and death by schistosomiasis mansoni [12,95,96]. Due to this phenomenon, the modification of the oviposition pattern, as well as the reduction in a load of eggs deposited in the tissues, indicate a better prognosis for the patient.

**Figure 4:**
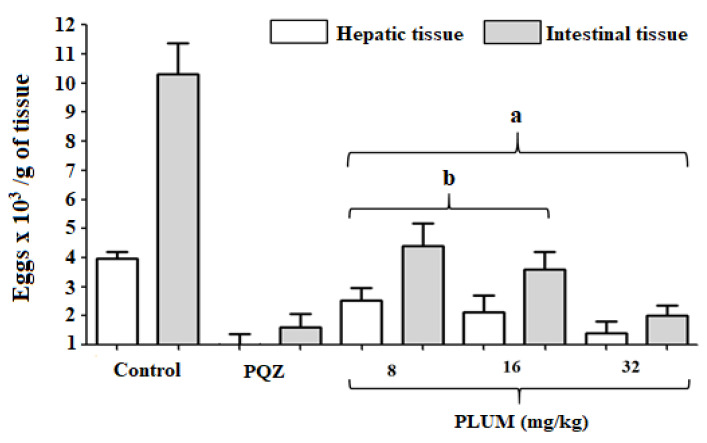
Effects of plumbagin (PLUM) and praziquantel (PZQ) on the load of eggs deposited in hepatic and intestinal tissues of mice infected with *Schistosoma mansoni*. ^a^ *p* < 0.001 compared to the control group hepatic and intestinal tissues and ^b^ *p* < 0.001 compared to the same type of tissue, hepatic or intestinal, with the group of animals treated with PZQ.

All groups treated with PLUM or PZQ showed a significant reduction *(p* < 0.001) in the load of eggs in the hepatic and intestinal tissues compared to the control group (Figure 4). When the treatment was carried out at doses of 8, 16, or 32 mg/kg, the reduction in eggs in the hepatic tissue was 36.11, 46.46, and 64.14%, and in the intestinal tissue, it was 57.22, 65.18, and 80.5%, respectively. PZQ reduces the egg load in the liver and intestines by 74.49 and 84.38%, respectively. Treatment with PLUM at a concentration of 32 mg/kg showed no statistical difference when compared to PZQ (Figure 4), indicating a similar effect to the drug recommended by the WHO. CO-ArNp reduced by 76.92 and 74.04% the presence of eggs in the hepatic and intestinal tissues, respectively. The authors argue that the strong activity against *S. mansoni* females directly affects the reduction to the complete absence of eggs in the tissues [81]. The results obtained by El-Beshbishia et al. [81] showed a greater reduction in the number of eggs deposited in the liver, while the reduction in the intestine was slightly lower than our results, although we highlight that the dosage of CO-ArNp (500 mg/kg) was much higher than those of PLUM. Our results are also in line with Aires et al. [50] in which *β*-lap (50 mg/kg) was also able to reduce the egg load in the liver tissue by 40.22% when compared to the control group and Bakery et al. [65] in which PLUM was able to reduce the liver load by 69.39% and the intestinal load by 68.79%.

### 3.2. Effects of PLUM on the Numerical and Volumetric of Schistosomal Hepatic Granulomas

**Figure 5:**
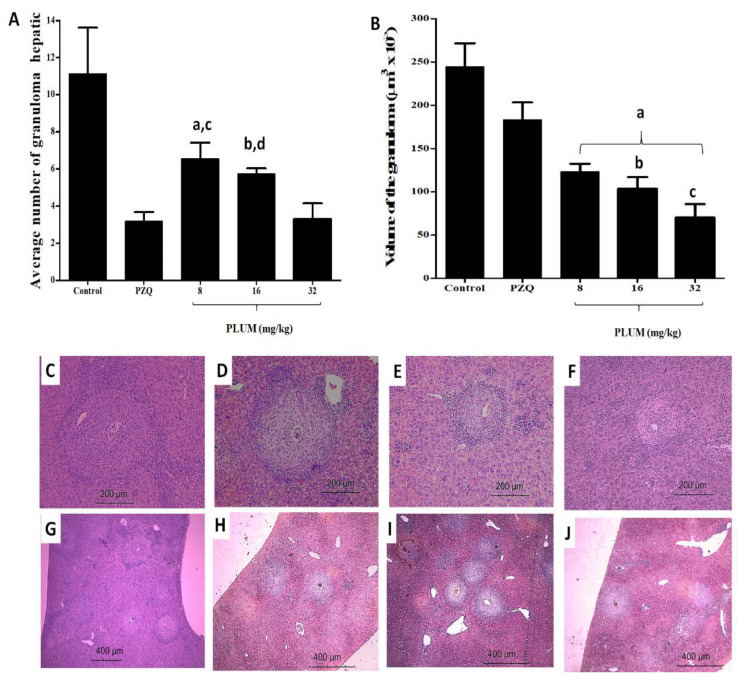
(**A**–**J**) Effects of plumbagin (PLUM) and praziquantel (PZQ) on hepatic schistosomal granuloma. (**A**,**B**) numerical density and volume of granulomas, respectively. In (**C**) Control group—showing periovular hepatic granuloma with intense infiltration of eosinophils, lymphocytic infiltrate with few thin collagen fibers (200×). (**D**)—treatment with pZQ exhibited an exudative granulomatous reaction, well-defined limits, little lymphocytic infiltrate and concentric collagen fibers distributed in the area of necrosis (200×). (**E**,**F**)—PLUM at doses of 16 and 32 mg/kg, respectively. In both groups, the granulomas exhibited reduced eosinophils and neutrophils and increased surrounding lymphocytes and macrophages, progressing to an isolated periovular productive stage, not exhibiting coagulative necrosis. In (**G**–**J**), the control, PZQ and PLUM groups are shown at doses 16 and 32 mg/kg, respectively; where it is possible to identify the reduction in density, size, and changes in the composition and organization of components and cells of the granulomatous inflammatory infiltrate.

Treatment with PLUM significantly reduced (*p* < 0.001) the mean number of hepatic granulomas by 41.11%, 48.47%, and 70.55% at doses of 8, 16, and 32 mg/kg, respectively, (Figure 5A) and PZQ reduced by 71.54%, with no statistical difference when compared to treatment at a dose of 32 mg/kg of PLUM (Figure 5A).

Histological samples from untreated infected mice (control) exhibited typical exudative periovular granulomatous schistosomal inflammation distributed in the hepatic parenchyma, characterized by an intense infiltrate of eosinophils and neutrophils, in addition to some macrophages and lymphocytes circumscribed to the living or partially degenerated, central egg (Figure 5C). Furthermore, some fields exhibited coalesced granulomas, foci of central coagulative necrosis, and dense and irregular collagen fibers distributed at the edge of the granuloma. Hepatic tissue from animals treated with PZQ exhibited an exudative granulomatous reaction, with more evident eggs, granuloma with well-defined limits, little lymphocytic infiltrate, and deposition of well-organized, dense concentric collagen fibers distributed in the area of necrosis (Figure 5D). On the other hand, PLUM caused a dose-dependent regression of the inflammatory reaction. Granulomas exhibited reduced eosinophils and neutrophils and increased surrounding lymphocytes and macrophages. The granuloma evolved to an isolated periovular productive stage, where the majority did not have a live egg, but only a shell or a trace of a central egg (Figure 5E,F). Despite the similarity in cellular composition, the doses of 16 and 32 mg/kg stood out for having finer collagen fibers, loosely organized and distributed concentrically to the granuloma, in addition to not exhibiting coagulative necrosis. This alteration in the composition and cell reorganization in the granulomas was able to reduce (*p* < 0.001) its volume by 49.56, 57.63, and 71.21% when the animals were treated with doses of 8, 16, and 32 mg/kg of PLUM, respectively, while PZQ reduced by only 25.01% (Figure 5B, vs. Figure 5G–J). The reduction in granuloma volume for the 32 mg/kg PLUM dose was significantly greater than PZQ (*p* < 0.001), and 8 (*p* < 0.001) and 16 (*p* < 0.01) mg/kg PLUM doses.

To numerical density: ^a^ *p* < 0.001 and ^b^ *p* < 0.01 compared to the PZQ group, ^c^ *p* < 0.001 and ^d^ *p* < 0.01 compared to the 32 mg/kg group of PLUM;

To volume of granulomas: ^a^ *p* < 0.001 compared to the control and PZQ groups, ^b^ *p* < 0.001 and ^c^ *p* < 0.01 compared to the 32 mg/kg group of PLUM.

The percentage reduction achieved with the PLUM treatment was greater than that obtained with CO-ArNp, which reduced the number and diameter of granulomas by 44.41% and 39.27% at a dose of 500 mg/kg, showed a reduction in granulomatous infiltration, more circumscription of granulomas, and egg degeneration when compared to the PZQ group. Furthermore, CO-ArNp also induced high rates of granuloma healing. The mechanisms proposed by the authors for this resolution of granulomas were the release of free radicals and an immunomodulatory effect; the same is true for other naphthoquinones, including PLUM. In this sense, Aires et al. [50] attribute a reduction in the size of schistosomal granuloma and improvement in histopathology to b-lapachone through its ability to inhibit neutrophil migration and reduce the synthesis of TNF-α, IL-6, NO, and NO- synthesize. Kapadia et al. [82] also identified a reduction in granuloma volume in the livers of infected mice treated with MEN, this time associating its effects with the reduction in type III procollagen peptides and inflammatory and immune responses in a redox-dependent manner. Bakery et al. [65], when evaluating the action of PLUM on schistosomal granuloma, demonstrated a reduction of 62.23% in the size of the granuloma, where the majority presented a central focus of mononuclear and polymorphonuclear cells around an egg, and the laminated layers of fibrotic tissue were minimal or had disappeared. Findings corroborate our study since PLUM reduced collagen fibers, altered cellular organization, and coagulative necrosis was not visualized, unlike treatment with PZQ, which, although it reduces the number of granulomas, reduces their volume by only 25%.

As far as is known, there are no studies in the clinical phase to evaluate new candidates for schistosomal drugs, despite the incentive for research and several studies, in vitro and in vivo, reporting a high rate of parasitological cure and favorable immunomodulation in granulomatous lesions. Mimicking the results achieved in experimental laboratory research in studies with human beings is challenging beyond ethical issues, since, although the results are promising, aspects such as solubility, absorption, bioavailability, and drug toxicity must be considered. It is known that naphthoquinones have limited solubility and absorption, in addition to being considered toxic.

### 3.3. In Silico Evaluation of Pharmacokinetic Parameters: Absorption, Distribution, Metabolism, Excretion, Toxicity (ADMET) and Prediction of Oral Bioavailability

The in silico prediction of ADMET and bioavailability parameters allows an assessment of the pharmacokinetic and toxicological profile of synthetic and natural compounds [75]. It is a quick and easy-to-perform study that assists in the selection of possible drug candidates [76]. Table 2 presents the prediction results for the ADMET and bioavailability parameters (Lipinski and Veber rules), predicted by the pkCSM and SwissADME platforms, respectively.

The first profile evaluated in Table 2 was absorption. The absorption of a drug depends on several factors and the first parameter to be evaluated was the solubility in water (LogS). For a drug to be absorbed, it needs to be dissolved in biological fluids [72]. Through the LogS values, it is possible to classify different compounds as insoluble < −10, slightly soluble < −6 <, moderately soluble < −4 <, soluble < −2 <, and very soluble < 0 [72]. PLUM and PZQ were classified as soluble.

Permeability was evaluated against Caco-2 cells (human colorectal adenocarcinoma cells). This prediction is carried out with the aim of verifying whether a given compound is capable of permeating intestinal cells [75,97]. PLUM and PZQ were considered moderately permeable (1 to 10.10^−6^ cm·s^−1^) [75].

Furthermore, as expected, high intestinal absorption was observed for PLUM 96.25% and high skin permeability (logKp < −2.5) [75]. Regarding skin permeability, both compounds can present high permeability (logKp < −2.5) [75]. Finally, it was evaluated whether PLUM and PZQ could be classified as substrates and/or inhibitors of p-glycoprotein and its isoforms. These glycoproteins act as an efflux pump against xenobiotics, protecting the body from the action of certain drugs, preventing the entry of drugs into the cell, or promoting their elimination, depending on their location [75]. 

In general, plumbagin can be classified as a non-substrate of p-glycoprotein, not being able to inhibit p-glycoprotein I (a drug transporter) and p-glycoprotein II (related to the biliary efflux of phosphatidylcholine) isoforms. Differently, PZQ can act as substrates. However, they are not inhibitors of glycoproteins I and II (Table 2).

The second profile to be predicted was the distribution. The volume of distribution (VDss) indicates the theoretical volume that a total dose would need to be uniformly distributed in plasma at the same concentration as observed in blood plasma. Compounds that present VDss values (log VDss) <−0.15 are more easily distributed in plasma and those that present VDss values (log VDss) > 0.45 are distributed in tissues [75]. There has been a trend that PLUM and PZQ is distributed in tissues. In addition, PLUM strongly binds to proteins with a low unbound fraction (Table 2). Regarding permeability in the blood–brain barrier (BB), compounds with logBB values > 0.3 easily cross the blood–brain barrier while compounds with logBB < −1 are poorly distributed to the brain [75]. Both molecules have the ability to cross the BB. Furthermore, PLUM and PQZ have a tendency to penetrate the CNS of the central nervous system [75].

The third profile evaluated was the metabolism to verify if PLUM and PZQ were able to act as inhibitors and substrates of cytochrome P450 and its isoforms. These enzymes promote the chemical modification of various exogenous lipophilic molecules, which then become more soluble and easily excreted by the human body [94]. PLUM was considered a non-substrate of CYP2D6 and CYP3A4 isoforms, while PZQ was considered a substrate for the latter. Furthermore, both molecules (PLUM and PZQ) were inhibitors only of the CYP1A2 and CYP3A4 isoforms, respectively (Table 2). Rocha et al. [98] reported that imidazoles isolated from Pilocarpus microphyllus acted to inhibit CYP2C19, CYP2C9, and CYP3A4. This inhibition reduces the ability of proteins to metabolize other drugs in the body, which suggests the possible accumulation of these metabolites favoring an improvement in relation to the pharmacological effect.

Regarding excretion (the fourth profile), PLUM showed a low clearance value, in addition to not being a renal OCT2 substrate when compared to PZQ. The fifth pattern evaluated was toxicity. The PLUM, different from PZQ, showed a positive result for the AMES test indicating a possible mutagenic effect. The recommended maximum tolerated dose (MRTD) was also investigated. Compounds that present an MRTD less than or equal to 0.477 log(mg/kg/day) are considered low and high if greater than 0.477 log(mg/kg/day). PLUM and PZQ showed a low MRTD; in addition, it is not able to inhibit hERG I and hERG II. It has moderate to high acute and chronic toxicity; however, it is neither hepatotoxic nor skin irritant. Drugs that have a schistosomicidal action and are predicted to be non-hepatotoxic become important molecules since the infection affects the liver [12,96]. PLUM and PZQ were considered toxic (pIGC_50_ > −0.5 log µg/L) against *T. Pyriformis*. In relation to minnow, log LC_50_ values < −0.3 are considered of high toxicity, therefore, we can classify PLUM and PZQ as low toxicity (Table 2). Finally, it was verified that PLUM obeys the rules of Lipinski and Veber indicating good oral bioavailability. Through in silico analysis, it was possible to verify that plumbagin can act as a promising drug candidate.

**Figure 6:**
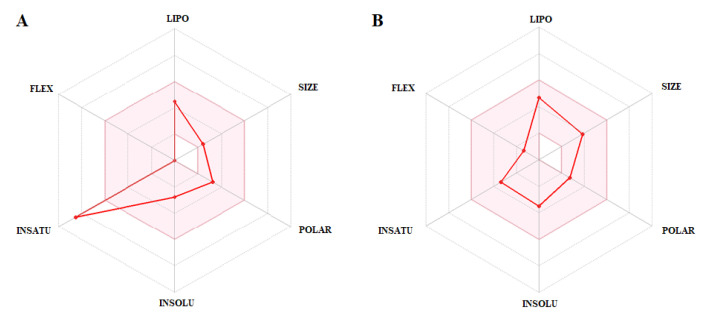
Bioavailability analysis: radar plots for the compounds, (**A**), plumbagin (PLUM) and (**B**), praziquantel (PZQ). Legend: LIPO (lipophilicity) = XLOGP3; SIZE (molecule size); POLAR (polarity) = TPSA; INSOLU = (insolubility); INSATU = (insaturation); fraction of carbons in sp3; FLEX = (flexibility).

PLUM and PZQ compounds were also analyzed according to the Bioavailability Radar graph (Figure 6) generated by the SwissADME platform, which correlates molecule size, flexibility, solubility, lipophilicity, saturation, and polarity. The pink area delimits the ideal bioavailability conditions for the administration of a drug orally [76]. The PLUM compound showed overestimated insaturation value (only one violation), that is, outside the area of a desirable profile. Finally, PZQ showed a good oral availability profile. These results show that the compounds evaluated have the potential to be used orally.

## 4. Conclusions

The results of this study demonstrate that PLUM is a promising schistosomal agent since it acted on parasitological parameters and granulomatous inflammation. Furthermore, we suggest that PLUM should be explored in new dosages, in pharmaceutical formulations, as a coadjuvant associated with PZQ to improve its activities and establish this drug as part of the available arsenal of schistosomicidal compounds. Finally, the study showed that PLUM has been present as a promising antischismosomal drug, contributing to a discovery of new therapeutic targets with acceptable pharmacokinetic profiles.

## Figures and Tables

**Table 1:** Effects of plumbagin (PLUM) and praziquantel (PZQ) on worm burdens (total and female) and oogram of mice infected with *S. mansoni*.

Experimental Groups	Average Worm Burden				% Of Eggs per Developmental Stage		
	Total	Reduction (%)	Female	Reduction (%)	Immature *	Mature	Dead
Control	32.14 ± 2.73	-	14.29 ± 1.6	-	53.99 ± 5.81	41.39 ± 5.88	4.57 ± 0.61
PZQ (50 mg/kg)	0.8 ± 0.44 ^a^	97.51	0.4 ± 0.54 ^a^	97.2	0.0 ± 0.0 ^g^	2.37 ± 1.4	97.63 ± 1.4 ^h^
PLUM							
8 mg/kg	17.13 ± 1.72 ^a^	46.7	8.0 ± 1.63 ^a^	44.01	29.67 ± 3.2 ^g^	32.33 ± 5.31	38.33 ± 4.41 ^h^
16 mg/kg	14.38 ± 1.84 ^a,b^	55.25	6.75 ± 0.89 ^a^	52.76	25.57 ± 3.64 ^g^	35.0 ± 55	39.43 ± 6.29 ^h^
32 mg/kg	8.87 ± 1.12 ^a,c,d^	72.4	4.12 ± 0.99 ^a,e,f^	71.16	18.83 ± 4.53 ^g^	37.0 ± 6.54	44.17 ± 7.41 ^h^

^a^ *p* < 0.001 compared to the respective control, total worms, or female worms; ^b^ *p* < 0.1; ^c^ *p* < 0.001 compared to the total worm load at the 8 mg/kg dose; ^d^ *p* < 0.001 when compared to the total burden of worms at the dose of 16 mg/kg; ^e^ *p* < 0.001 when compared to the burden of female worms at a dose of 8 mg/kg; ^f^ *p*< 0.1 when compared to the total load of worms at a dose of 16 mg/kg; ^g^ *p*< 0.001 compared to the percentage of immature eggs in the control group; ^h^ *p*< 0.001 compared to the percentage of dead eggs in the control group.

**Table 2:** ADMET parameters and evaluation of the oral bioavailability of plumbagin (PLUM) and praziquantel (PZQ).

Parameters	PLUM	PZQ	Unit
Absorption			
Water solubility	−2.65	−4.00	Numeric (log mol/L)
Caco2 permeability	1.19	1.75	Numeric (log Papp in 10^−6^ cm/s)
Intestinal absorption	96.25	93.42	Numeric (%Absorbed)
Skin Permeability	−2.93	−3.14	Numeric (log Kp)
P-glycoprotein substrate	No	Yes	Categorical (Yes/No)
P-glycoprotein I inhibitor	No	No	Categorical (Yes/No)
P-glycoprotein II inhibitor	No	No	Categorical (Yes/No)
Distribution			
VDssa	0.14	0.52	Numeric (log L/kg)
Fraction unbound	0.48	0.15	Numeric (Fu)
BBB permeability	0.47	0.46	Numeric (log BB)
CNS permeability	−2.82	−1.78	Numeric (log PS)
Metabolism			
CYP2D6 substrate	No	No	Categorical (Yes/No)
CYP3A4 substrate	No	Yes	Categorical (Yes/No)
CYP1A2 inhibitor	Yes	No	Categorical (Yes/No)
CYP2C19 inhibitor	No	Yes	Categorical (Yes/No)
CYP2C9 inhibitor	No	No	Categorical (Yes/No)
CYP2D6 inhibitor	No	No	Categorical (Yes/No)
CYP3A4 inhibitor	No	No	Categorical (Yes/No)
Excretion			
Total clearance	0.14	1.05	Numeric (log mL/min/kg)
Renal OCT2 substrate	No	Yes	Categorical (Yes/No)
Toxicity			
AMES toxicity	Yes	No	Categorical (Yes/No)
Maximum tolerated dose	0.40	−0.23	Numeric (log mg/kg/day)
hERG I inhibitor	No	No	Categorical (Yes/No)
hERG II inhibitor	No	No	Categorical (Yes/No)
Oral rat acute toxicity	1.63	2.26	Numeric (mol/kg)
Oral rat chronic toxicity	2.55	1.11	Numeric (log mg/kg_bw/day)
Hepatotoxicity	No	No	Categorical (Yes/No)
Skin sensitization	No	No	Categorical (Yes/No)
*T. Pyriformis* toxicity	0.71	1.31	Numeric (log µg/L)
Minnow toxicity	1.75	1.56	Numeric (log mM)
Oral bioavailability			
Lipinski’s rule	0	0	Violation (numeric)
Veber’s rule	0	0	Violation (numeric)

BBB: Blood–Brain Barrier; CNS: Central Nervous System; CYP: Cytochrome P450; hERG: Human Ether-a-go-go Related Gene; OCT2: Organic cation transporter 2; *T. pyriformis*: *Tetrahymena pyriformis*.

## Data Availability

The data presented in this study are available on request from the corresponding author.
